# A Non-Stationary Geometry-Based MIMO Channel Model for Terahertz UAV-Based Wireless Communication Systems

**DOI:** 10.3390/e28070744

**Published:** 2026-07-01

**Authors:** Zican Jiang, Yongjun Li, Kai Zhang, Jianguo Liu

**Affiliations:** 1 School of Information and Navigation, Air Force Engineering University, Xi’an 710082, China; mallowmerlin@163.com (Z.J.); 13262733921@163.com (K.Z.); 2 Institute of Semiconductors, CAS, Beijing 100083, China

**Keywords:** terahertz (THz), non-stationary, UAV channel modeling, geometry-based MIMO channel model, THz UAV channels, scattering cluster, ISAC

## Abstract

UAV-assisted communication is widely regarded as a key component of next-generation Space-Air-Ground Integrated Networks (SAGINs), where integrated sensing and communication (ISAC) further drives the demand for accurate and reliable channel modeling. Terahertz (THz) communications are particularly attractive for UAV platforms, offering ultra-high data rates and physically secure transmission. However, the physical heterogeneity between reflection and scattering mechanisms in THz UAV channels poses significant modeling challenges, as conventional unified approaches tend to introduce energy distribution distortion and non-stationary prediction errors. To address this, we propose a 3D non-stationary geometry-based stochastic model (GBSM) based on an ellipse-sphere hierarchical geometric framework, where reflection paths are confined to ground-plane ellipses and scattering paths are distributed over spatial spheres. The model accounts for atmospheric molecular absorption, multipath fading, and non-stationarity induced by random 3D UAV trajectories. A cluster birth-death mechanism is introduced to capture the time-varying evolution of scattering clusters. Key statistical properties, including the temporal auto-correlation function (T-ACF), spatial cross-correlation function (S-CCF), and Doppler power spectral density (DPSD), are derived and analyzed. Simulation results agree well with theoretical derivations, validating the proposed model and providing practical guidance for THz UAV-ISAC system design.

## 1. Introduction

### 1.1. Motivation

As sixth-generation (6G) mobile communication technology becomes the foundation of future communication, utilizing the terahertz (THz) band for ultra-high-speed wireless transmission has emerged as a primary research focus [1]. Compared to the peak data rates of tens of THz in 5G networks, 6G systems are expected to support rates exceeding 1 Tb/s by exploiting the abundant bandwidth of the Hz spectrum and spatial diversity [2]. To achieve seamless global coverage, 6G wireless networks will evolve from traditional terrestrial infrastructures into Space-Air-Ground-Sea Integrated Networks (SAGIN), incorporating multi-tier architectures such as satellites, unmanned aerial vehicles (UAVs), and terrestrial ultra-dense networks. As key enablers for 6G, both Sag-sins and THz communications have naturally gained significant traction. However, realizing these novel architectures and their performance targets requires a thorough understanding of the wireless channel characteristics specific to 6G technologies. Accurate channel modeling remains the basis for system design, network optimization, and performance evaluation, making dedicated channel research for 6G networks highly critical. While existing channel models can partially characterize the lower THz bands by updating specific attenuation parameters (e.g., molecular absorption coefficients), they face significant bottlenecks at frequencies above 300 GHz, particularly in UAV scenarios. Conventional models tend to utilize a unified statistical distribution to characterize all multipath components (MPCs). However, at >300 GHz, the severe propagation sparsity amplifies the physical heterogeneity between ground reflections and spatial scattering. Merely adapting parameters within conventional unified frameworks fails to capture this physical divergence, often leading to severe energy distribution distortion. Therefore, rather than a simple parametric extension, a fundamentally novel geometric framework is required to structurally isolate and independently evolve these heterogeneous signal components under high-mobility conditions.

### 1.2. Related Work

#### 1.2.1. UAV-Based Channels

As integral components of the Space-Air-Ground-Sea Integrated Networks (SAGINs) in the 6G era, Unmanned Aerial Vehicles (UAVs) serving as aerial nodes have emerged as indispensable platforms for extending network coverage and enhancing capacity. Currently, the integration of high-speed 3D mobility into both infrastructure and terminal devices introduces pronounced channel non-stationarity, which in turn drives the urgent demand for high-throughput services in various drone applications and compels continuous advancements in drone channel modeling. To satisfy these rigorous high-throughput demands and mitigate complex fading effects in dynamic airborne environments, Multiple-Input Multiple-Output (MIMO) technology has been widely incorporated into UAV communication systems [3,4]. Fundamentally, for a generic UAV-MIMO system equipped with P transmit and Q receive antennas, the spatial multiplexing and transmission process can be mathematically formulated as $r\left(t\right)=H(t)*s(t)+n(t)$. Here, $s(t)\in C^{P\times 1}$, $r(t)\in C^{Q\times 1}$, and $n(t)\in C^{Q\times 1}$ denote the transmitted signal, received signal, and channel noise vectors, respectively. The term $H(t)\in C^{Q\times P}$ represents the time-variant channel impulse response (CIR) matrix, which fundamentally dictates the capacity limits and is the core subject of our channel modeling. Precise spatio-temporal channel characterization is fundamental to the design of efficient UAV systems, necessitating modeling approaches that are generally categorized into deterministic and stochastic methodologies. Among these, deterministic models—such as Ray Tracing (RT)—a deterministic approach that simulates high-frequency propagation paths based on geometrical optics [5,6,7]—and Finite-Difference Time-Domain (FDTD) [8,9] methods, which numerically solve Maxwell’s equations over discretized spatial grids and offer high accuracy but incur prohibitive computational complexity, rely on detailed and exhaustive descriptions of the propagation environment, which inevitably leads to prohibitive computational complexity. Conversely, stochastic models evaluate channel parameters statistically. At the same time, while Non-Geometric Stochastic Models (NGSMs) [10,11] distribute effective scatterers according to specific statistical laws, they lack a universal geometric structure. Consequently, they often fail to provide a conceptual framework that can be meaningfully generalized across varying and complex propagation scenarios.

**Figure 1:**
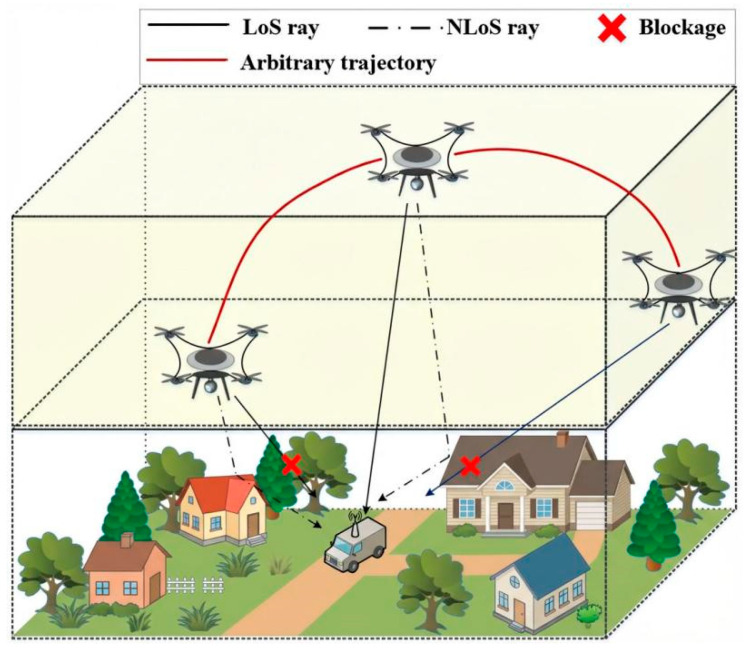
Typical UAV-based THz A2G wireless communications scenario in the suburbs.

To achieve a practical trade-off between computational efficiency and modeling flexibility, Geometry-Based Stochastic Models (GBSMs) [12,13,14,15] have been widely adopted for characterizing UAV channels. GBSMs physically map random scatterers onto simplified spatial shapes (e.g., spheres, cylinders, or ellipsoids), making them highly suitable for reproducing the spatial-temporal non-stationarity of UAV communications. To date, most studies have focused on the microwave and millimeter-wave bands [16,17,18]; however, research specifically investigating non-stationary UAV channels at frequencies above 100 GHz remains notably scarce. Although GBSMs are extensively employed in the literature across various UAV scenarios, the majority of existing models are specifically tailored for conventional communication systems. For this reason, their established results cannot be directly generalized to the unique propagation characteristics of Terahertz (THz) systems. This is particularly relevant for THz communications, which depend heavily on Line-of-Sight (LoS) propagation. A significant limitation of current GBSMs is their tendency to utilize a unified statistical distribution to characterize all multipath components (MPCs). Such an oversimplified treatment overlooks the inherent physical heterogeneity of different signal components, potentially leading to inaccurate performance evaluations.Therefore, we need to conduct channel measurement and channel model design for models in different scenarios, as shown in Figure 1.

#### 1.2.2. THz Channel in UAV-MIMO Systems

Owing to its ultra-wide bandwidth and exceptionally high data rates, the terahertz (THz, 0.1–10 THz) band has been identified as one of the core spectral resources for future 6G systems and beyond, with additional potential as a carrier for high-precision wireless sensing. Nevertheless, THz signal propagation differs substantially from that observed in the microwave and millimeter-wave bands. Specifically, THz signals are subject to more severe frequency-selective fading, heightened sensitivity to atmospheric molecular absorption, and greater susceptibility to blockage and scattering effects [19]. These distinctive propagation characteristics make the development of accurate THz channel models a fundamental prerequisite for advancing research in this field.

Existing studies consistently indicate that research on THz wireless communications remains in its early stages. To gain a thorough understanding of THz channel propagation behavior, extensive measurement campaigns have been carried out across various research groups worldwide [16,17,18,19,20,21,22,23,24,25,26]. Early investigations primarily focused on short-range indoor environments and fixed point-to-point links, with path loss typically predicted through Fresnel zone approximations or Ray Tracing (RT) techniques, supplemented by molecular absorption line models such as the HITRAN database [27,28] to characterize absorption peaks and frequency-dependent attenuation. A series of measurement campaigns and channel analyses targeting specific deployment scenarios—including urban microcells (UMi), vehicular-to-everything (V2X) networks, and indoor office environments—have been reported in [20,21,22,23,24,25,26], collectively providing valuable empirical evidence and data support for THz channel modeling and revealing scenario-specific propagation mechanisms. A comprehensive survey of THz wireless channels, covering measurement methodologies, modeling approaches, and the associated analysis of open issues and future research directions for 6G THz systems, is presented in [22].

It is further worth noting that, given the high carrier frequency and wide bandwidth of THz channels, non-stationary behavior becomes considerably more pronounced compared to lower frequency bands. In [18], a generic three-dimensional space-time-frequency non-stationary GBSM is proposed for 6G THz ultra-massive MIMO systems, demonstrating the ability to capture channel properties across diverse scenarios including indoor, device-to-device (D2D), ultra-massive MIMO, and long-path propagation links. In [29], a three-dimensional time-varying channel model that jointly accounts for small-scale fading and molecular absorption is developed for THz UAV dual-mobility links.

To date, a substantial body of measurement campaigns and channel modeling work [14,15,16,17,18,19,20,21,22,23,24,25,26,27,28,29,30,31,32,33,34,35,36,37,38,39,40,41,42,43,44,45,46,47] has been dedicated to THz wireless channels, with propagation characteristics analyzed across a range of studies. However, the majority of these efforts have concentrated on frequency bands below 300 GHz, while channel characteristics above 300 GHz remain comparatively underexplored and poorly understood, underscoring the need for dedicated future measurement campaigns in this regime. As a result, channel models and propagation characteristics specifically tailored to THz UAV MIMO wireless communications remain insufficiently defined. Further in-depth investigation is warranted, encompassing more comprehensive channel modeling frameworks, systematic wideband measurements, and rigorous performance analyses.

### 1.3. Contributions

In this study, we compare our work against conventional stationary 3D Geometry-Based Stochastic Models (GBSMs) and traditional unified scattering frameworks—which do not strictly differentiate the physical heterogeneity between reflection and scattering mechanisms in THz channels. As previously discussed, when directly applied to highly mobile UAV scenarios, these existing approaches tend to introduce energy distribution distortion and non-stationary prediction errors. To overcome the limitations of these reference models, our proposed framework explicitly distinguishes these mechanisms and captures time-varying characteristics to achieve higher accuracy. Motivated by gaps in current research, this paper proposes a 3D GBSM for non-stationary THz UAV channels. The main contributions and innovations of this paper are summarized as follows:

1. This paper proposes a 3D non-stationary geometric MIMO channel model for THz UAV communications, which features a stratified multi-path structure combining an ellipsoid model and a two-sphere model.
2. To account for the non-stationarity resulting from the mobility of the Tx, Rx, and clusters, we incorporate a cluster birth-death process into the scattering parts of NLOS components, thereby enhancing the representation of spatial non-stationarity.
3. To gain a deeper understanding of the channel characteristics, we simulate and analyze key statistical properties, including the Space-Time-Frequency Correlation Function (STF-CF) and the Doppler Power Spectrum (DPS).

Based on these statistics, we present several insightful and practical observations that facilitate channel tracking and the establishment of reliable UAV communication links.

## 2. Three-Dimensional Non-Stationary UAV-MIMO Channel Model

In this paper, we consider a UAV-based THz MIMO communication system where the transmitter (Tx) and receiver (Rx) are equipped with antenna arrays composed of isotropic antenna elements. In a generic UAV-Ground Station MIMO communication system, the antennas at the Tx and Rx are located at the UAV and on the ground station, respectively, and the numbers of antennas at the Tx and Rx are P and Q, respectively. The UAV is equipped with P transmit antennas and is capable of hovering and moving in three-dimensional space, while the ground terminal is equipped with Q receive antennas and positioned at a certain height. In the proposed 3D GBSM framework, the specific characteristics of the Uniform Linear Arrays (ULAs), such as antenna spacing and array orientation, are implicitly captured by assigning distinct 3D spatial coordinate vectors to each antenna element. Consequently, the spatial phase shifts between different antenna elements are naturally determined by the geometric distances from these coordinate vectors to the 3D scattering clusters (i.e., the ellipse-sphere framework. Since UAVs typically execute complex maneuvers such as climbing, diving, and circling, the direction of the transmit velocity vector must be characterized using both azimuth and elevation angles). $\theta_{Tx}$ and $\phi_{Tx}$ characterize the azimuth and elevation of the transmitter’s direction of motion, respectively. As for the ground terminals, since they move solely on the ground, the direction of their velocity can be simply described by the azimuth angle $\theta_{Rx}$ and elevation angle ${\;\phi }_{Rx}$.

**Figure 2:**
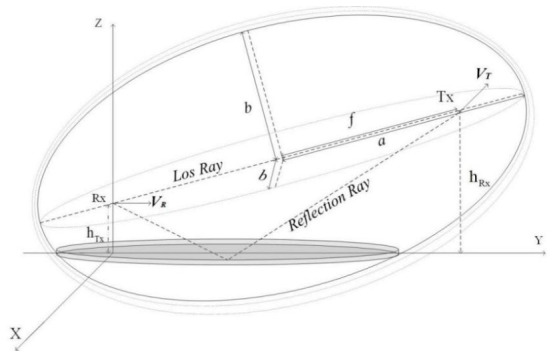
The LOS path and the reflection paths in the time-varying UAV-based A2G wireless communication system in the THz band.

**Figure 3:**
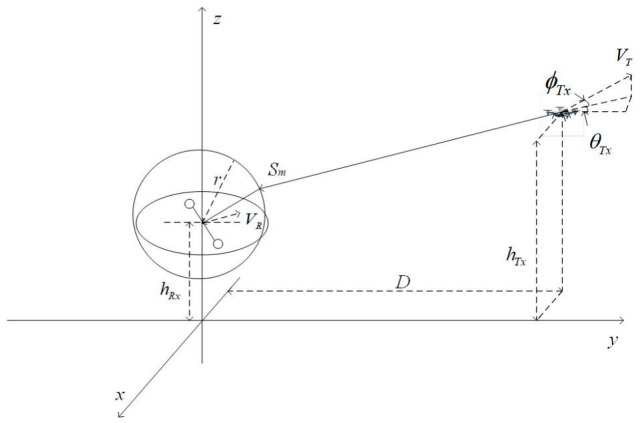
The scattering paths in the time-varying UAV-based A2G wireless communication system in the THz band.

UAV-based communication is characterized by random 3D deployment, random 3D trajectories, flexible environmental adaptability, and high spatiotemporal variability. For such spatiotemporally varying channels, multipath propagation arises from free-space transmission, reflection off obstacles, and scattering from rough surfaces. Accordingly, the propagation paths are divided into Line-of-Sight (LoS) and Non-Line-of-Sight (NLoS) components. Aiming for both precision and efficiency in characterizing THz UAV channels, this scheme differentiates multipath sources into two categories. In terahertz UAV channel modeling, this paper adopts a hierarchical modeling strategy that explicitly categorizes multipath signal sources into three independent components: line-of-sight (LoS), specular reflection, and scattering, which are modeled separately. This design stems from the physical nature of wireless propagation in the terahertz band. Existing geometry-based stochastic models typically employ a unified statistical distribution to describe all multipath components, a “one-size-fits-all” approach that severely neglects the physical heterogeneity of signal components. Specular reflection occurs on smooth surfaces, follows the laws of geometrical optics, and exhibits highly concentrated energy with predictable paths, whereas scattering occurs on rough surfaces, with energy diffusing in all directions and exhibiting strong randomness. The extremely short wavelength of terahertz waves (millimeter scale) causes the same surface to potentially generate both strong reflection and strong scattering simultaneously. Traditional volumetric scattering models distribute all multipath components randomly in 3D space, leading to the non-physical phenomenon of “airborne suspended reflectors.” By strictly confining reflectors to the intersection curve between the ellipsoid surface and the ground plane, and distributing scatterers on a sphere surrounding the receiver, this paper effectively circumvents this issue. Moreover, although reflection paths are fewer in number, they carry 15–30% of the total energy, while scattering paths, though more numerous, account for only 5–10% of the energy. This energy magnitude difference necessitates different precision strategies. The hierarchical modeling enables the space-time correlation function of the total channel to be decomposed into a linear superposition of independent components, greatly simplifying theoretical derivation and providing differentiated optimization strategies for system design, while reducing computational complexity by approximately 50% without sacrificing accuracy. Therefore, this hierarchical modeling approach is essential for addressing the propagation characteristics of the terahertz band. As shown in Figure 2, LoS and specular reflection paths are modeled using 3D confocal ellipsoids, while scattering components are captured by a spherical geometric framework in Figure 3.

With the severe propagation path loss in A2G THz links—including spreading loss, scattering and reflection fading, and atmospheric absorption—we consider only single-bounce reflections for the NLoS paths, as multi-bounce components are negligible. As shown in Figure 2, a Cartesian coordinate system with the origin O is established. Let $\left(x_{p}(t),y_{p}(t),z_{p}(t)\right)$ and $\left(x_{q}(t),y_{q}(t),z_{q}(t)\right)$ denote the spatial coordinates of the p-th (p = 1, …, p) antenna element at the transmitter (Tx) and the q-th (q = 1, …, q) antenna element at the receiver (Rx) at time t separately. Furthermore, the antenna element spacings of the Tx and Rx arrays are defined as $d_{T}$ and $d_{R}$, respectively.

In this scenario, we assume that the Rx is initially located at the origin of the coordinate system, and that reflection fading from rough surfaces occurs exclusively on the Rx side. Reflectors obey Snell’s law with deterministic geometric constraints, whereas scatterers originate from rough surface interactions and are inherently stochastic. A unified treatment would obscure these distinct propagation mechanisms. Therefore, distinguishing them facilitates compressed sensing (CS)-based sparse channel estimation and enables decoupled optimization—specifically, beamforming for reflectors and diversity combining for scatterers. As shown in Figure 3, $S_{m}$ denotes the m-th scatterer (m = 1, …, M) around the Rx, which is located on the surface of a sphere centered at the Rx. Here, M represents the number of effective scatterers. Meanwhile, multiple confocal ellipsoids with the Tx and Rx as foci constitute a delay-line structure. The effective reflection points are uniformly distributed along the confocal ellipses formed by the intersection of these ellipsoidal surfaces and the ground plane. The n-th reflector is denoted by $R_{n}$ (n = 1, …, N). N represents the number of effective reflectors. Since the UAVs at both the Tx and Rx are in continuous motion, the coordinates of the reflector $R_{n}$ and the scatterer $S_{m}$ are separately denoted as $\left(x_{n}(t),y_{n}(t),z_{n}(t)\right)$ and $\left(x_{m}(t),y_{m}(t),z_{m}(t)\right)$. Other parameters of the UAV-based terahertz (THz) communication system are detailed in Table 1. It should be emphasized that the ellipses and ellipsoids defined in this framework represent delay-based spatial boundaries rather than Fresnel zones. Specifically, for a ground-plane reflection ellipse, the foci are the projections of the Tx and Rx on the ground plane, separated by a distance 2f. The major axis (2a) is determined by the specific path delay τ (2a = c∙τ, where c is the speed of light), and the minor axis (2b) is given by $2b=2\sqrt{a^{2}-f^{2}}$. By modeling the initial phases of the paths originating from these regions as uniformly distributed random variables over [0, 2*π*), the model intrinsically captures both constructive and out-of-phase (destructive) multipath interference. Similarly, the spheres in the proposed framework define the effective spatial boundaries of scattering clusters. Unlike the delay-constrained reflection ellipses, the radius r of a scattering sphere represents the spatial spread of the cluster. The maximum boundary of the sphere, $r_{max}$, is determined by the intra-cluster delay spread ($\Delta \tau_{cluster}$) obtained from empirical THz measurements, where $r_{max}\propto c\cdot \Delta \tau_{cluster}$. The precise radial distance rr of any specific scatterer within the sphere is stochastically generated following an exponential distribution bounded by $r_{max}$. This statistical modeling of r effectively captures the realistic spatial density variations in atmospheric and local scatterers. While physical reflecting surfaces such as the ground form a continuous set, standard GBSM practices approximate these continuous regions using a finite number of discrete effective sub-paths, denoted as N. This discretization allows for computationally tractable channel simulations while preserving the accurate statistical approximation of the fading envelope. Furthermore, while the ground ellipse captures the dominant delay-specific ground bounce, reflections and scattering from 3D environmental elements (e.g., building surfaces) are comprehensively encapsulated within the spatial scattering spheres. The rationale and distinct superiority of employing the hybrid ellipse-sphere geometric framework, rather than a unified single-geometry model (such as a pure ellipsoid or a pure sphere), lie in its capability to physically decouple the heterogeneous propagation mechanisms inherent in THz UAV air-to-ground (A2G) links. At frequencies above 300 GHz, the propagation environment exhibits extreme sparsity, which amplifies the physical divergence between ground reflections and airborne scattering. Ground reflections are predominantly specular and geometrically restricted to the horizontal ground plane, which is naturally modeled by ground-plane ellipses. In contrast, airborne scattering from spatial obstacles (such as buildings, foliage, or atmospheric particles) is diffuse and omnidirectional, which is best captured by 3D spatial spheres. Utilizing a single geometry would force these disparate components to share the same spatial-temporal statistics, resulting in severe angular and delay distortions. Furthermore, this hybrid structure enables the decoupling of their temporal non-stationarity: ground-reflected paths are relatively stable and long-lived, whereas spatial scattering clusters undergo fast birth-death processes due to the rapid 3D motion of the UAV. By separately modeling these heterogeneous components, the proposed framework accurately preserves the physical energy distribution and time-varying Doppler characteristics, yielding a significantly more realistic channel representation.

Based on wireless transmission theory, the received signal $r\left(t\right)={\left[r_{1}(t),\;r_{2}(t)\ldots ,\;r_{Q}(t)\right]}^{T}$ of an MIMO system is determined by the CIR matrix $H\left(t\right)={\left[h_{qp}\left(t\right)\right]}_{Q\times P}(q\;$= 1, …, Q; p = 1, …, P), the transmitted signal $s\left(t\right)={\left[s_{1}(t),\;s_{2}(t)\ldots ,{\;s}_{P}(t)\right]}^{T}$, and the channel noise $n\left(t\right)={\left[n_{1}(t),{\;n}_{2}(t)\ldots ,{\;n}_{Q}(t)\right]}^{T},$ which can be formulated as $r\left(t\right)=H(t)*s(t)+n(t)$.

In the time-variant wireless communication system described herein, the CIR between the p-th transmit antenna (p = 1, …, P) and the q-th receive antenna (q = 1, …, Q) can be further expressed as a function of the following time-variant parameters.

(1) $$h_{qp}\left(t,\tau \right)=\sum_{l=1}^{L(t)}\left[\alpha_{i}(t)e^{j\Phi_{i}(t)}\right]\cdot \delta (\tau -\tau_{l}(t))$$

where $\alpha_{i}(t)$ denotes the time-variant channel gain, $\tau_{2}(t)$ denotes the time-variant propagation path delay, $f_{c}$ denotes the carrier frequency, and $f_{D,l}(t)$ denotes the time-variant Doppler frequency shift.

Meanwhile, this paper considers the Line-of-Sight (LoS) path, the reflected path, and the scattered path as relatively independent parts. The CIR can be formulated as

(2) $$h_{qp}\left(t,\tau \right)=h_{qp}^{LoS}(t,\tau )+h_{qp}^{ref}(t,\tau )+h_{qp}^{sca}(t,\tau )$$

In UAV-assisted three-dimensional (3D) non-stationary communication systems, the high mobility of the UAV results in its flight altitude exhibiting continuous and dynamic time-variant characteristics. Meanwhile, given that the wavelength of Terahertz (THz) waves is significantly shorter than that of millimeter-wave (mmWave) and microwave bands, THz signals are more sensitive to the surface texture of the propagation environment. Consequently, when interacting with rough surfaces of different materials, THz waves demonstrate more significant scattering and diffuse reflection effects compared to lower frequency bands. With the increase in operating frequency, the atmospheric molecular absorption attenuation coefficient, $A_{ma}(f_{c},t)$, rises significantly, becoming a critical factor limiting the distance of Terahertz (THz) communication [48]. Due to the susceptibility of THz propagation to the medium, the Channel Impulse Response (CIR) model constructed in this paper primarily focuses on environmentally induced path loss. Consequently, the model emphasizes quantifying the scattering effects from rough surfaces and the atmospheric absorption attenuation caused by water vapor molecular resonance. Furthermore, it is assumed that the antennas are isotropic, and the directional gain provided by beamforming technology is not considered.

## 3. Channel Parameters’ Generation and Time Evolution

Based on the model as described before, this paper classifies the propagation paths into three categories: the Line-of-Sight (LoS) path, the reflected path, and the scattered path.

### 3.1. LoS Path

As shown in Figure 3, in the time-variant Line-of-Sight (LoS) propagation channel described in this paper, there are no obstacles between the transmitter and the receiver. In this case, the Channel Impulse Response (CIR) of the time-variant LoS path between the p-th element of the transmit antenna array and the q-th element of the receive antenna array can be expressed as:

(3) $$h_{qp}^{LoS}\left(t\right)=\sqrt{\frac{\Omega_{qp}(t)K_{qp}(t)}{K_{qp}(t)+1}}\cdot e^{j\left\{2\pi t\left(f_{D,LoS,p}^{Tx}(t)+f_{D,LoS,q}^{Rx}(t)\right)-\frac{2\pi d_{n,qp}^{LoS}(t)}{\lambda_{c}}\right\}}$$

Here, $d_{LOS,p}(t)$ denotes the 3D distance between the p-th transmit antenna element and the q-th receive antenna element along the LoS path. $\lambda =c/f_{c}$ is the radio wavelength, where c is the speed of light. The Rician factor $K_{qp}(t)$ pertains to the wireless propagation link that exists between the p-th antenna element of the transmitting antenna array and the q-th antenna element of the receiving antenna array. In this paper, it is assumed that $K_{qp}(t)$ =${\;K}_{qp}$. Finally, $\Omega_{qp}\left(t\right)=E[{|h_{qp}(t)|}^{2}$] indicates the propagation path power from the p-th antenna element of the transmitting antenna array and the q-th antenna element of the receiving antenna array, where E[⋅] denotes the statistical expectation operation.

In Equation (3),${\;f}_{D,LoS,p}^{Tx}(t)$ and $f_{D,LoS,q}^{Rx}(t)$ denote the Doppler frequency shifts at the p-th element of the transmit antenna array and the q-th element of the receive antenna array, respectively. Their expressions are given by:

(4) $$f_{D,LoS,p}^{Tx}\left(t\right)=f_{Tm}\left[\cos\left(\theta_{LoS,p}^{AoD}(t)-\theta_{Tx}\right)\cdot \cos\left(\phi_{LoS,p}^{AoD}(t)\right)\cos(\phi_{Tx})+\sin\left(\phi_{LoS,p}^{AoD}(t)\right)\sin(\phi_{Tx})\;\right]$$

(5) $$f_{D,LoS,q}^{Rx}\left(t\right)=f_{Rm}\left[\cos\left(\theta_{LoS,q}^{AoA}(t)-\theta_{Rx}\right)\cdot \cos\left(\phi_{LoS,q}(t)\right)\right]$$

### 3.2. Reflection Path

The Non-Line-of-Sight (NLoS) propagation model constructed in this paper primarily consists of two components: reflection and scattering. In terms of geometric modeling, a three-dimensional (3D) ellipsoid model and a spherical scattering model are employed to characterize the propagation properties of the reflected and scattered paths, respectively. Based on the above assumptions, this study considers only single-bounce paths. Regarding the spatial distribution of effective scatterers, it is assumed that the effective scattering points near the receiver (Rx) follow a uniform random distribution. As for the reflection links, the effective reflection points are assumed to be randomly distributed along the confocal elliptical trajectory formed by the intersection of the 3D ellipsoid and the ground plane.

Given the Cartesian coordinates of the transmitter (Tx), receiver (Rx), and the reflection point, one can calculate the propagation distance of the reflected signal from the p-th element of the transmit antenna array to the q-th element of the receive antenna array at the n-th reflection surface. The expression is given by:$\;d_{nqp}^{ref}\left(t\right)=\left|d_{Tx,np}^{ref}(t)\right|+\left|d_{Rx,qn}^{ref}(t)\right|$. Here, $d_{Tx,np}^{ref}(t)$ and $d_{Rx,qn}^{ref}(t)$ denote the propagation distances involving the n-th reflection surface, the p-th transmit antenna element, and the q-th receive antenna element.

According to the description above, the CIR of the time-variant reflection path between the p-th transmit antenna element and the q-th receive antenna element is given by

(6) $$h_{qp}^{ref}(t)=\sqrt{\frac{\Omega_{qp}(t)\eta_{ref,qp}(t)}{N[K_{qp}(t)+1]}}\sum_{n=1}^{N}\alpha_{n,qp}^{ref}(t)e^{j\vartheta_{ref,n}}\times e^{j\left[2\pi t\left(f_{D,n,p}^{Tx,ref}(t)+f_{D,q,n}^{Rx,ref}(t)\right)-\frac{2\pi d_{n,qp}^{ref}(t)}{\lambda_{c}}\right]}$$

The Doppler shift terms $f_{D,np}^{Tx,ref}(t)$ and $f_{D,qn}^{Rx,ref}(t)$ are defined as follows:

(7) $$f_{D,np}^{Tx,ref}\left(t\right)=f_{Tn}\left[\cos\left(\theta_{ref,np}^{AoD}(t)-\theta_{Tx}\right)\cos\left(\phi_{ref,np}^{AoD}(t)\right)\cos\left(\phi_{Tx}\right)+\sin(\phi_{ref,np}^{AoD}(t))\sin(\phi_{Tx})\right]$$

(8) $$f_{D,qn}^{Rx,ref}\left(t\right)=f_{Rn}\left[\cos\left(\theta_{ref,qn}^{AoA}(t)-\theta_{Rx}\right)\cdot \cos(\phi_{Rx})\right]$$

Here, $f_{Tn}$ and $f_{Rn}$ denote the distinct Doppler frequency shifts induced by the motion of the Tx and Rx for the nn-th path, respectively. Unlike the direct LoS path where Doppler is governed by pure relative speed, the Doppler shift for scattered NLoS paths must be calculated piece-wise. The velocities of the Tx and Rx ($v_{T}$ and $v_{R}$) are integrated into the maximum Doppler frequencies $f_{Tn}\;$=${\;v}_{T}$/λ, and $f_{Rn\;}$=$\;v_{R}$/λ.

In modeling the spatial topology of reflection paths, we strictly limit the reflector distribution to the intersection of the 3D confocal ellipsoid and the ground plane to ensure physical authenticity. This assumption combines geometric theory with environmental constraints: geometrically, the ellipsoid (with Tx and Rx as foci) represents the iso-delay surface; physically, given the sensitivity of Terahertz waves, strong specular reflectors (e.g., concrete pavements) are invariably located on the ground (z = 0) rather than floating randomly in space. Thus, the intersection trajectory represents both the mathematical iso-delay curve and the only valid physical region for reflections. This geometric constraint-based approach avoids the unrealistic artifacts of ‘suspended reflectors’ typical of traditional volumetric models, thereby improving the physical fidelity of the non-stationary UAV channel’s joint statistical characteristics in the angular and delay domains. Accordingly, the equation for the ground intersection ellipse is given by:

(9) $$\frac{(x-x_{off}{)}^{2}}{a^{2}}+\frac{y^{2}}{b^{2}}=1$$

where

(10) $$a=\frac{x_{max}^{\prime }-x_{min}^{\prime }}{2\sin\varphi }$$

(11) $$b=\left|\sqrt{a^{\prime 2}-\frac{a^{\prime 2}}{b^{2}}{\left[x_{off}^{\prime }\cot\varphi -f^{\prime }-\frac{h_{Tx}}{\sin\varphi }\right]}^{2}-(x_{off}^{\prime }{)}^{2}}\right|$$

where $a^{\prime }$, $b^{\prime }$, and $f^{\prime }$ denote the semi-major axis, semi-minor axis, and focal length of the 3D ellipsoid, respectively. Meanwhile,$\;f^{\prime }$= D/2. $h_{Tx}$ represents the height of the receive antenna, while $x_{max}^{\prime }$ and $x_{min}^{\prime }$ indicate the coordinate values of the two endpoints (extrema) of the intersection ellipse along the major axis within the 3D ellipsoidal coordinate system.

### 3.3. Scattering Path

As scatterers are uniformly distributed around the receiver, the CIR for the time-variant scattering path between the p-th transmit element and the q-th receive element is given by:

(12) $$\begin{array}{ll} h_{qp}^{sca}\left(t\right) & =\sqrt{\frac{\Omega_{qp}(t)\eta_{sca,qp}(t)}{K_{qp}(t)+1}}\\ & \times \sum_{l=1}^{L(t)}\frac{w_{l}(t)}{\sqrt{L(t)M_{l}}}\sum_{m=1}^{M_{l}}\alpha_{m,qp}^{sca}(t)e^{jJ_{sca,m}}\\ & \times e^{\left\{2j\pi t\left(f_{D,m_{p}}^{Tx,sca}(t)+f_{D,qm}^{Rx,sca}(t)\right)-\frac{2\pi d_{m,qp}^{sca}(t)}{\lambda_{c}}\right\}} \end{array}$$

$w_{l}(t)$ denotes the birth-death factor of the cluster, used to weight the contribution of the l-th cluster and reflect the power distribution among different clusters. $M_{l}$ represents the number of sub-paths (scattering elements) within the l-th cluster, corresponding to the count of discrete multipath components inside the scatterer. L(t) indicates the number of ‘active’ (or ‘surviving’) scattering clusters at time t, which varies over time governed by the birth-death mechanism.

$f_{D,mp}^{Tx,sca}(t){,\;f}_{D,qm}^{Rx,sca}(t)$ can be respectively expressed as:

(13) $$f_{D,mp}^{Tx,sca}\left(t\right)=f_{Tm}\left[\cos\left(\theta_{sca,mp}^{AoD}(t)-\theta_{Tx}\right)\cos\left(\phi_{sca,mp}^{AoD}(t)\right)\cos\left(\phi_{Tx}\right)+\sin(\phi_{sca,mp}^{AoD}(t))\sin(\phi_{Tx})\right]$$

(14) $$f_{D,qm}^{Rx,sca}\left(t\right)=f_{Rm}\left[\cos\left(\theta_{sca,qm}^{AoA}(t)-\theta_{Rx}\right)\cos\left(\phi_{sca,qm}^{AoA}(t)\right)\cos\left(\phi_{Rx}\right)+\sin(\phi_{sca,qm}^{AoA}(t))\sin(\phi_{Rx})\right]$$

### 3.4. Dynamic Cluster Set Evolution Mechanism

To characterize the time-varying nature of the scattering environment in non-stationary channels, this paper introduces a dynamic cluster set evolution mechanism. This mechanism models the realistic turnover of multipath components during UAV movement. Here, L(t) denotes the number of ‘active’ (or ‘surviving’) scattering clusters at time t. This quantity exhibits discrete time-variant jumps and is dynamically governed by a birth-death mechanism. The set of active scattering clusters, C(t), is updated according to geometric and probabilistic birth-death rules:

(15) $$L\left(t\right)=L(t-\Delta t)+L_{birth}(t)-L_{death}(t)$$

where $L_{birth}\;(t)$ represents the number of newly generated scattering clusters within the time interval Δt, following a Poisson distribution $P(\lambda_{G}\Delta t)$ with the parameter $\lambda_{G}\Delta t$. $\lambda_{G}$ denotes the cluster generation rate, and $L_{death}(t)$ represents the number of clusters removed via the survival probability decision mechanism. For a random l-th scattering cluster, its survival probability at the next time instant, denoted as $P_{surv,l}(t)$, is defined as:

(16) $$P_{surv,l}\left(t\right)=P_{geo,l}(t)\cdot e^{-\lambda_{R}\Delta t}$$

**Figure 4:**
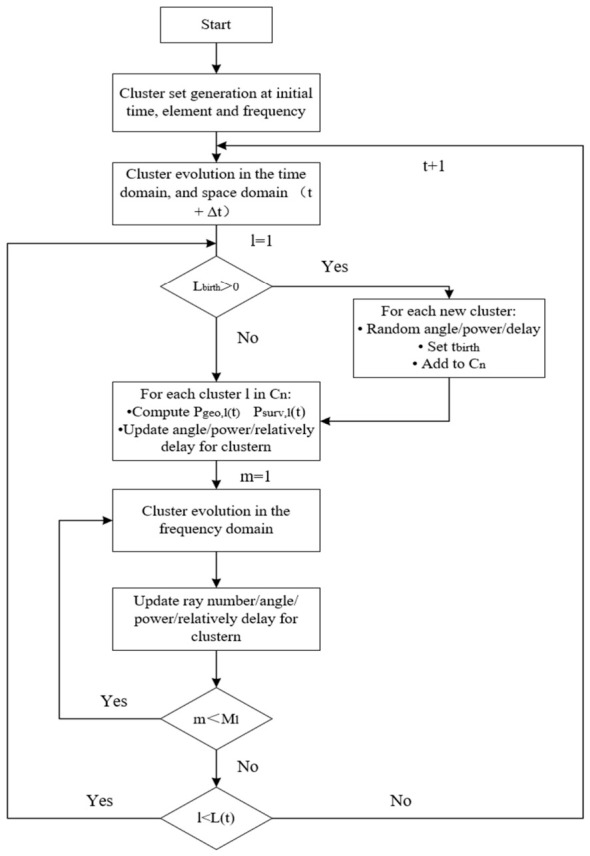
The standard birth-death mechanism decision of clusters in the three-dimensional GBSM model.

Here, $\lambda_{R}$ denotes the cluster removal rate, which characterizes the rate of dynamic changes in the scattering environment. $P_{geo,l}\left(t\right)\;$∈ {0, 1}, which is a binary geometric indicator representing whether the cluster is within the physically visible line-of-sight region, serves as a geometric visibility indicator, used to determine whether the l-th scattering cluster lies within the visible range of the transceiver antennas at the current time instant. This mechanism ensures that the model can dynamically simulate the realistic evolution of multipath components during UAV motion, thereby accurately reflecting the time-varying nature of non-stationary channels. Practically, the survival decision of a cluster at each time step is executed by generating a continuous uniform random variable X∼U(0,1). If ${X<P}_{surv,l}$, the cluster survives; otherwise, it is removed. The process is shown in Figure 4.

However, the direct cluster birth-death mechanism leads to non-physical step discontinuities in the time-domain cluster power profile, which consequently introduces artificial high-frequency spectral leakage and Gibbs phenomenon artifacts into the time-variant Doppler spectrum. To ensure the temporal continuity and physical plausibility of the channel energy, this paper introduces a smoothing weighting function $w_{l}(t)$ to modulate the amplitude of the complex gain for each cluster. $w_{l}(t)$ denotes the birth-death factor of the l-th cluster, which is used to weight the contribution of the l-th cluster. It reflects the evolution of power distribution for different clusters within their lifespans. The specific mathematical formulation of $w_{l}(t)$ is determined by the lifecycle status of the cluster. Here, $t_{birth,l}$ and $t_{death,l}$ denote the time instants when the l-th cluster is identified by the algorithm as generated (birth) and vanished (death), respectively, while ∆T represents the preset length of the smoothing transition window. The weighting function is defined as:

(17) $$w_{l}\left(t\right)=\left\{\begin{array}{ll} \sin^{2}\left(\frac{\pi (t-t_{birth,l})}{2\Delta T}\right), & t_{birth,l}\;\leq \;t\;\leq {\;t}_{birth,l}\;+\;\Delta T\\ 1, & t_{birth,l}\;+\;\Delta T\;\leq \;t\;\leq \;t_{death,l}\\ \cos^{2}\left(\frac{\pi (t-t_{death,l})}{2\Delta T}\right), & t_{death,l}\;\leq \;t\;\leq {\;t}_{death,l}\;+\;\Delta T\\ 0, & t\;\geq \;t_{death,l}\;+\;\Delta T \end{array}\right.$$

In the birth phase, $w_{l}(t)$ guides the energy of the new cluster to increase smoothly from zero to the maximum value following a sine-squared curve, thereby achieving a gradual injection of energy; in the stable phase, the weight remains at 1, allowing the cluster to contribute to the channel response with full power. In the Removal Phase, $w_{l}(t)$ utilizes a cosine-squared curve to smoothly attenuate the energy of the old path from its maximum value to zero, achieving a gradual energy fade-out; in the death phase, the weight returns to zero. Mathematically, this design eliminates step discontinuities in the impulse response, ensures the continuity of $w_{l}(t)$ and its first derivative, and effectively suppresses high-frequency oscillatory artifacts in the Doppler spectrum. For the l-th cluster, $M_{l}$ denotes the number of sub-paths (scattering sub-elements), corresponding to the count of discrete multipath components within the scatterer. The complex gain of each sub-path is uniformly modulated by $w_{l}(t)$, thereby ensuring that the energy contribution of the entire cluster exhibits smooth transition characteristics in the time domain. By integrating the dynamic cluster set evolution mechanism with the energy smoothing transition weighting function, the proposed model achieves physical continuity in the channel’s time-varying process while maintaining the accuracy of statistical properties. This establishes a theoretical foundation for the high-fidelity modeling of non-stationary UAV channels.The mechanism is shown as Figure 4. The single-headed arrows represent the progression of the process.

### 3.5. Important Parameters

Based on the empirical attenuation model for oxygen and water vapor molecules in ITU-R P.676-11, Kirchhoff scattering theory, the modified Beckmann–Kirchhoff theory, and the Friis transmission equation, the channel coefficients for the reflection and scattering components can be calculated as follows:

(18) $$\alpha_{n,qp}^{ref}\left(t\right)=\frac{\frac{\lambda_{c}^{2}\cdot R_{n,qp}(f_{c},t)\cdot a_{ma,n,qp}^{ref}(f_{c},t)}{16\pi^{2}d_{T_{x},np}^{ref}(t)d_{Rx,qn}^{ref}(t)}}{\sqrt{\frac{1}{N}{\sum_{n=1}^{N}\left[\frac{\lambda_{c}^{2}\cdot R_{n,qp}(f_{c},t)\cdot a_{ma,n,qp}^{ref}(f_{c},t)}{16\pi^{2}d_{T_{x},np}^{ref}(t)d_{Rx,qn}^{ref}(t)}\right]}^{2}}}$$

(19) $$\alpha_{m,pq}^{sca}\left(t\right)=\frac{\frac{\lambda_{c}^{2}\cdot S_{m,pq}(f_{c},t)\cdot a_{ma,m,pq}^{sca}(f_{c},t)\cdot w_{I}(t)}{16\pi^{2}d_{Tx,mp}^{sca}\cdot d_{Rx,qm}^{sca}(t)}}{\sqrt{\frac{1}{L(t)M_{l}}\sum_{l=1}^{L(t)}{\sum_{m=1}^{M_{l}}\left[\frac{\lambda_{c}^{2}\cdot S_{m,qp}(f_{c},t)\cdot a_{ma,m,qp}^{sca}(f_{c},t)\cdot w_{l}(t)}{16\pi^{2}d_{Tx,mp}^{sca}(t)\cdot d_{Rx,qm}(t)}\right]}^{2}}}$$

where $R_{n,qp}(f_{c},t)$ and $S_{m,qp}(f_{c},t)$ denote the reflection and scattering propagation path coefficients on the rough surface for the link from the p-th element of the transmit antenna array to the q-th element of the receive antenna array, respectively; $a_{ma,n,qp}^{ref}(f_{c},t)$ and $a_{ma,m,qp}^{sca}(f_{c},t)$ represent the channel gains resulting from atmospheric absorption along the reflection and scattering paths, respectively. The specific formulas are given by:

(20) $$a_{ma,m,qp}^{ref}\left(f_{c},t\right)=e^{-\frac{1}{2}\int_{0}^{t_{np,q}^{ref}(t)}k_{surf}(f_{c})\cdot e^{-\frac{h(l)}{H}}dl}$$

(21) $$a_{ma,m,qp}^{sca}\left(f_{c},t\right)=e^{-\frac{1}{2}\int_{0}^{t_{np,q}^{sca}}k_{surf}(f_{c})\cdot e^{-\frac{h(l)}{H}}dl}$$

$k_{surf}(f_{c})$ is the molecular absorption coefficient at the surface (altitude h = 0). H represents the scale height, which corresponds to the height where the absorption coefficient decreases to 1/e (approx. 37%) of the ground value. Finally, h(l) is the instantaneous altitude of the l during transmission.

## 4. Statistical Properties of the Proposed UAV-Based Terahertz Channel Model

Based on the 3D non-stationary GBSM constructed above, this section further theoretically derives the key statistical properties of the channel, namely the Space-Time Correlation Function (STCF) and the Doppler Power Spectral Density (DPSD).

In the derivation process, considering the distinct physical propagation mechanisms, it is assumed that the time-variant impulse responses of the Line-of-Sight (LoS), specular reflection, and scattering components are statistically independent. This implies that the random phases, amplitude fluctuations, and Doppler shifts within each component are mutually independent. Based on this independence assumption, the statistical correlation of the total channel can be expressed as the linear superposition of the correlations of the individual components. Therefore, for any two complex envelope sub-channels $h_{qp}(t)$ and $h_{q\prime p\prime }(t)$ in an MIMO system, the normalized STCF can be decomposed and calculated as follows [49]:

(22) $$\begin{array}{ll} R_{pq,p^{\prime }q^{\prime }}\left(d_{T},d_{R},t,\tau \right) & =\frac{E[h_{pq}(t)(h_{p\prime q\prime }(t+\tau ){)}^{*}]}{\sqrt{E[|h_{pq}(t){|}^{2}]E[|h_{p\prime q\prime }(t+\tau ){|}^{2}]}}\\ & =\frac{E\left[h_{pq}(t){\left(h_{p^{\prime }q^{\prime }}(t+\tau )\right)}^{*}\right]}{\sqrt{\Omega_{pq}(t)}\;\Omega_{p^{\prime }q^{\prime }}(t+\tau )} \end{array}$$

where E[·] and ${\left[\cdot \right]}^{*}$ represent the statistical expectation operator and the complex conjugate operation, respectively. In Equations (22)–(26), dT and dR denote the antenna element spacings of the transmitting antenna array and the receiving antenna array, respectively.

Based on multipath propagation theory [50], the total complex envelope of the Terahertz (THz) channel is composed of three distinct physical propagation mechanisms: Line-of-Sight (LoS), specular reflection from smooth surfaces, and diffuse scattering from rough surfaces. Given the fundamental differences in the physical nature of these mechanisms, this paper assumes that their corresponding multipath components are mutually independent in the phase, angle, and delay domains. This implies that there is no statistical correlation between components generated by different mechanisms, and the expected value of their cross-correlation terms is zero. Building upon this independence assumption and the principle of linear superposition, the Space-Time Correlation Function (STCF) of the total channel can be expressed as a linear superposition of the correlation functions for the LoS, specular reflection, and diffuse scattering components. This decomposition approach not only aligns with physical intuition but also provides a theoretical foundation for the subsequent independent modeling and parameter extraction of each mechanism, enabling the complex characteristics of the THz channel to be systematically characterized through the analysis of the statistical properties of individual propagation mechanisms. Thus, the equation can be expressed as:

(23) $$R_{pq,p^{\prime }q^{\prime }}\left(d_{T},d_{R},t,\tau \right)\;=R_{pq,p\prime q\prime }^{LoS}(d_{T},d_{R},t,\tau )+R_{pq,p\prime q\prime }^{ref}(d_{T},d_{R},t,\tau )+R_{pq,p\prime q\prime }^{sca}(d_{T},d_{R},t,\tau )$$

The individual components are given by:

(24) $$\begin{array}{l} R_{p,q,p^{\prime },q^{\prime }}^{LoS}\left(d_{T},d_{R},t,\tau \right)=\;\frac{E\left[h_{LoS,pq}(t){\left(h_{LoS,p\prime q\prime }(t+\tau )\right)}^{*}\right]}{\sqrt{\Omega_{pq}(t)\Omega_{p\prime q\prime }(t+\tau )}}\\ =\;\sqrt{\frac{K_{pq}(t)K_{p^{\prime }q^{\prime }}(t)}{\left(K_{pq}(t)+1)(K_{p^{\prime }q^{\prime }}(t)+1\right)}}\times e^{-\frac{2}{\lambda_{c}}\left[d_{LOS,pq}(t)-d_{LOS,p\prime q\prime }(t+\tau )\right]}\\ \times e^{j2\pi t\left[f_{D,LoS,p}^{Tx}(t)+f_{D,LoS,q}^{Rx}(t)\right]-j2\pi (t+\tau )\left[f_{D,LoS,p\prime }^{Tx}(t+\tau )+f_{D,LoS,q\prime }^{Rx}(t+\tau )\right]} \end{array}$$

(25) $$\begin{array}{l} R_{pq,p^{\prime }q^{\prime }}^{ref}\left(d_{T},d_{R},t,\tau \right)=\frac{E\left[h_{pq}^{ref}(t){\left(h_{p\prime q\prime }^{ref}(t+\tau )\right)}^{*}\right]}{\sqrt{\Omega_{pq}(t)\Omega_{p\prime q\prime }(t+\tau )}}\\ =\sqrt{\frac{\eta_{pq}(t)\eta_{p^{\prime }q^{\prime }}(t)}{\left(K_{pq}(t)+1)(K_{p^{\prime }q^{\prime }}(t)+1\right)}\cdot \sum_{n=1}^{N}\alpha_{n,pq}^{ref}(t)\cdot \alpha_{n,p^{\prime }q^{\prime }}^{ref}(t+\tau )}\\ \times e^{-j\frac{2\pi }{\lambda_{c}}[d_{n,pq}^{ref}-d_{n,p^{\prime }q^{\prime }}^{ref}(t+\tau )]}\times e^{j2\pi t[f_{D,nq}^{Tx,ref}(t)+f_{D,qn}^{Rx,ref}(t)]}\\ \times e^{-j2\pi (t+\tau )}\left[f_{D,np^{\prime }}^{Tx,ref}(t+\tau )+f_{D,q^{\prime }n}^{Rx,ref}(t+\tau )\right] \end{array}$$

(26) $$\begin{array}{ll} & R_{pq,p^{\prime }q^{\prime }}^{sca}\left(d_{T},d_{R},t,\tau \right)=\frac{E\left[h_{pq}^{sca}(t){\left(h_{p\prime q\prime }^{sca}(t+\tau )\right)}^{*}\right]}{\sqrt{\Omega_{pq}\Omega_{p\prime q\prime }(t+\tau )}}\\ & =\sqrt{\frac{\eta_{pq}(t)\eta_{p^{\prime }q^{\prime }}(t)}{\left(K_{pq}(t)+1)(K_{p^{\prime }q^{\prime }}(t)+1\right)}\sum_{l=1}^{L(t)}\frac{w_{l}(t)w_{l}(t+\tau )}{M_{l}\sqrt{L(t)L(t+\tau )}}}\\ & \times \sum_{m=1}^{M_{l}}\alpha_{m,pq}^{sca}(t)\cdot \alpha_{m,p\prime q\prime }^{sca}(t+\tau )\times e^{-j\frac{2\pi }{\lambda_{c}}[d_{m,pq}(t)-d_{m,p\prime q\prime }(t+\tau )]}\\ & \times e^{j2\pi t\left[f_{D,mp}^{Tx,sca}(t)+f_{D,qm}^{Rx,sca}(t)\right]-j2\pi (t+\tau )\left[f_{D,mp}^{Tx,sca}(t+\tau )+f_{D,qm}^{Rx,sca}(t+\tau )\right]} \end{array}$$

In Equations (16)–(20), $d_{T}$ and $d_{R}$ denote the antenna element spacings of the transmit and receive antenna arrays, respectively. Furthermore, by setting $d_{T}=d_{R}=0$ or τ=0, the Temporal Auto-Correlation Function (T-ACF) and the Spatial Cross-Correlation Function (S-CCF) can be obtained separately.

The DPSD can be derived by applying the Fourier transform to the T-ACF with respect to τ [51], and its expression is given by

(27) $$\begin{array}{l} S_{DPSD,qp,q^{\prime }p^{\prime }}\left(t,f_{D}\right)=S_{DPSD,qp^{\prime },q^{\prime }p^{\prime }}^{LoS}\left(t,f_{D}\right)+S_{DPSD,qp,q^{\prime }p^{\prime }}^{ref}\left(t,f_{D}\right)+S_{DPSD,qp,q^{\prime }p^{\prime }}^{sca}\left(t,f_{D}\right)\\ \;=\int_{-\infty }^{+\infty }R_{qp,q^{\prime }p^{\prime }}^{LoS}(t,\tau )e^{-j2\pi f_{D}\tau }\;d\tau +\int_{-\infty }^{+\infty }R_{qp,q^{\prime }p^{\prime }}^{ref}(t,\tau )e^{-j2\pi f_{D}\tau }\;d\tau +\int_{-\infty }^{+\infty }R_{qp,q^{\prime }p^{\prime }}^{sca}(t,\tau )e^{-j2\pi f_{D}\tau }\;d\tau \end{array}$$

## 5. Results and Analysis

In this section, we conduct numerical analyses of the proposed 3D time-variant UAV-MIMO channel model through statistical properties such as T-ACF, S-CCF, and DPSD, considering various channel parameters and carrier frequencies in the millimeter-wave and terahertz bands. To address the global scarcity of field-measurement data in the upper THz band for dynamic UAV links, deterministic ray-tracing (RT) simulations—which are strictly formulated on electromagnetic propagation physics (Geometrical Optics and diffraction theories)—are utilized to generate high-fidelity emulated measurement data. The model parameters are configured in close alignment with physical environment layouts and standardized databases (such as the HITRAN database for atmospheric attenuation). The proposed hierarchical GBSM is validated against these physics-based RT simulations. By comparing the statistical properties with both the RT benchmark and conventional unified models, the superior accuracy and physical consistency of the proposed model are rigorously demonstrated. According to [26], the parameters for our analysis are set as follows: P = Q = 2, $\alpha_{Tx}$ = $\alpha_{Rx}$ = $\beta_{Tx}$ = $\beta_{Rx}$ = 0°, $n_{t,ref}$ = $n_{t,sca}$ = 2.2 (concrete surface), $\eta_{ref,qp}$ = $\eta_{ref,q\prime p\prime }$ = 0.6, $\eta_{sca,qp}$ = $\eta_{sca,q\prime p\prime }$ = 0.4, $\sigma_{h,ref}$ = 0.05 mm, and $\sigma_{h,sca}$ = 0.15 mm, where $\sigma_{h,sca}$ represents the standard deviation of the rough surface height along the scattering path. The simulation parameter data is shown as Table 2.

### 5.1. ACF

**Figure 5:**
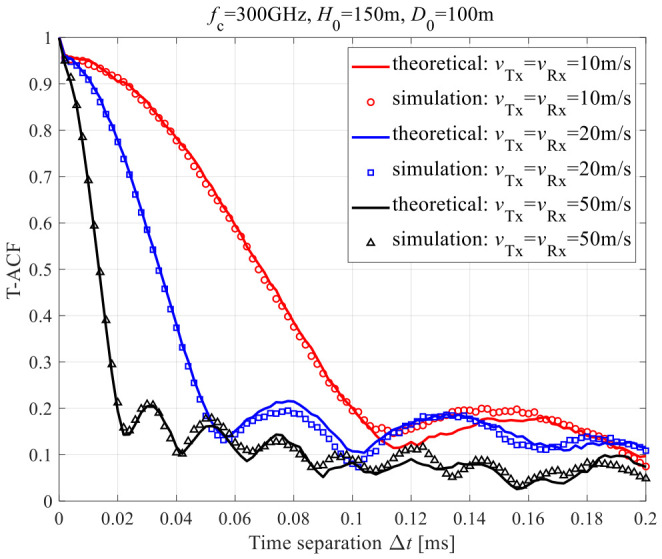
Simulation and theoretical results of the S-CCF for the NLoS path with different initial times.

Figure 5 illustrates the T-ACF for different Tx and Rx velocities and time intervals at t = 0 s, where $D_{0}$ are the initial vertical and horizontal distances between Tx and Rx. The theoretical curves match well with simulation results, validating the proposed model. It can be seen that the T-ACF decreases more rapidly as Tx/Rx velocities increase, in agreement with [12]. This is attributed to the proportional relationship between CIR phase variations and Tx/Rx velocities.

#### Vertical Distances Between Tx and Rx

**Figure 6:**
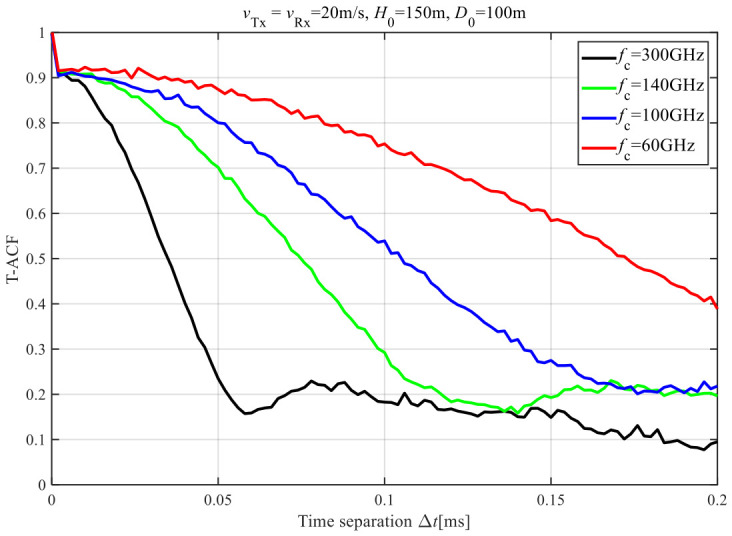
Theoretical T-ACF for NLOS paths at different carrier frequencies.

In Figure 6, the theoretical T-ACF of NLOS paths under various carrier frequencies are shown: 60 GHz, 140 GHz, 200 GHz, and 300 GHz. The results reveal that the T-ACF exhibits a decreasing trend with increasing frequency, demonstrating faster channel decorrelation in higher frequency bands. The T-ACF at 60 GHz remains relatively high, whereas it decreases substantially at 300 GHz, highlighting the fast time-varying nature of millimeter-wave and terahertz channels. Furthermore, the coherence time, determined at T-ACF = 0.8, is inversely proportional to the carrier frequency. Lower frequencies (e.g., 60 GHz) yield longer coherence times, while higher frequencies (200 GHz–300 GHz) result in significantly shorter coherence times, indicating rapid channel state transitions.

### 5.2. SCCF

**Figure 7:**
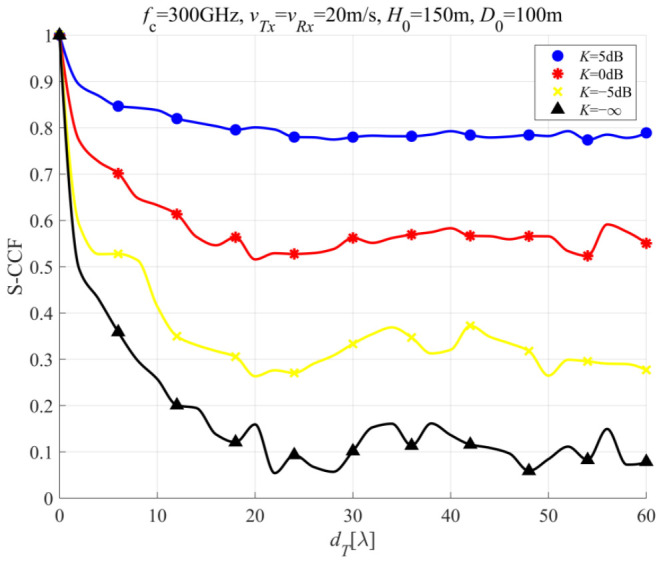
Theoretical results of the S-CCF for the LoS + NLoS path with different Rician factors.

Figure 7 illustrates the theoretical S-CCF of LoS + NLOS paths at t = 0 s for different Rician K-factors and transmit antenna element spacings. As observed, the S-CCF grows with increasing K-factor and exhibits strong dependence on antenna spacing. The Rician K-factor quantifies the power ratio between LoS and NLOS (reflection and scattering) paths. When K is large, the LoS path dominates, enhancing the deterministic signal component while suppressing multipath effects. This results in high spatial correlation among antenna elements and elevated S-CCF values. As K decreases, NLOS components gain prominence, introducing rich multipath from various angles with distinct delays and phases, thereby increasing channel randomness and reducing spatial correlation. In the limiting case where K → −∞ (pure Rayleigh channel), the LoS component is absent, scattering dominates, and S-CCF reaches its minimum. At K = 5 dB, the strengthened LoS component markedly raises the S-CCF curve. Moreover, antenna spacing impacts spatial correlation: reduced spacing increases correlation, while increased spacing decreases it, in accordance with classical theory.

**Figure 8:**
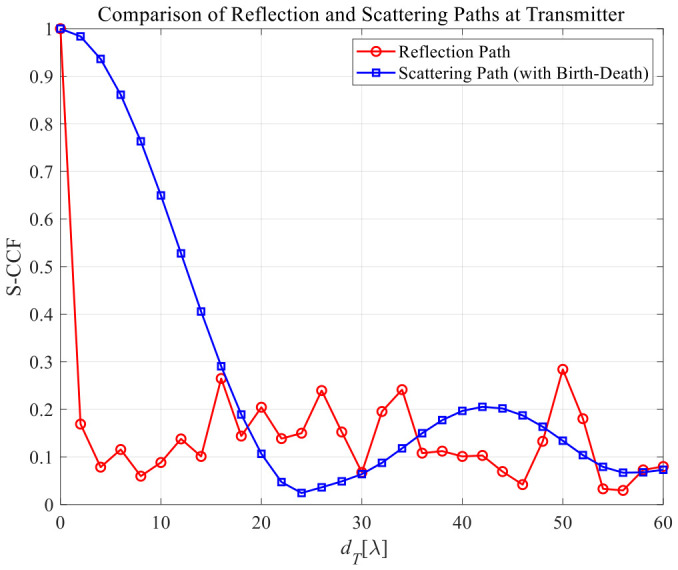
Theoretical results of the S-CCF for the reflection and scattering paths.

Figure 8 illustrates the spatial correlation comparison between reflection paths and scattering paths (incorporating the birth-death mechanism) at the transmitter side for demonstrating the superiority of the layered modeling approach. The two mechanisms display fundamentally different spatial correlation patterns. For reflection paths, the widespread distribution of ground reflection points leads to rapid spatial decorrelation: the S-CCF decreases sharply to approximately zero within 5λ antenna spacing, followed by periodic oscillations caused by constructive and destructive multipath interference at varying spacings. Scattering paths with the birth-death mechanism behave differently. Since scattering clusters concentrate in a limited area near the receiver, the S-CCF exhibits smooth monotonic decay from unity, retaining values above 0.6 even at 60λ spacing. From a geometric perspective, a UAV at 100 m altitude and 150 m horizontal distance observes the 5 m scattering region with an angular spread of merely ~1.6°, far smaller than the 37° spread subtended by the 75 m reflection region, accounting for the slower correlation decay. This pronounced difference validates the layered modeling approach: reflection and scattering represent distinct physical processes with fundamentally different spatial correlation properties. Conventional unified NLOS modeling cannot capture this duality. The proposed layered model, integrating the scattering cluster birth-death process, accurately quantifies individual mechanism contributions and provides flexibility to model diverse A2G propagation scenarios—from open ground to dense scattering environments—through power ratio parameterization.

### 5.3. DPSD

**Figure 9:**
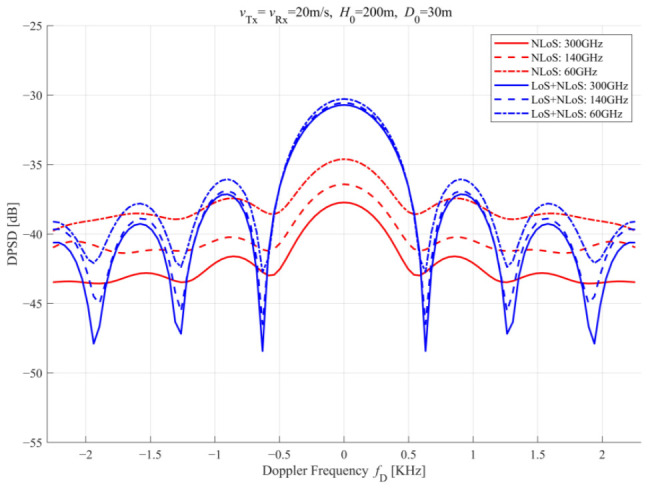
DPSD with different propagation paths (i.e., NLoS path and LoS + NLoS path) and carrier.

According to the proposed model, Figure 9 illustrates the theoretical Doppler power spectral density (DPSD) at t = 0 s under different carrier frequencies (i.e., 300 GHz, 140 GHz, and 60 GHz) and propagation paths (i.e., NLOS and LoS + NLOS).

As observed, NLOS paths (depicted by red curves) exhibit smooth bell-shaped spectra centered at zero Doppler frequency, with spectral width proportional to the carrier frequency. This frequency-dependent Doppler spreading originates from the wavelength sensitivity of angular scattering distributions: shorter wavelengths at higher frequencies provide enhanced angular resolution, resulting in broader spectral occupancy.

The DPSD magnitude increases with carrier frequency for both LoS + NLOS and NLOS paths. Notably, NLOS paths demonstrate significantly larger variations compared to LoS + NLOS paths. Furthermore, across all carrier frequencies examined, the DPSD dynamic range of LoS + NLOS paths consistently exceeds that of NLOS paths.

#### Frequencies

**Figure 10:**
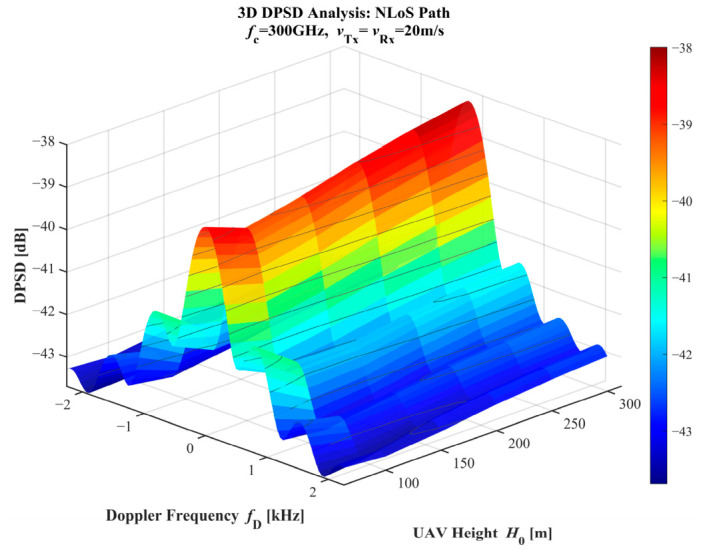
Theoretical results of the DPSD for the NLoS path with different UAV heights of Tx antenna arrays when the moving time t is 0 s.

Figure 10 depicts the three-dimensional Doppler power spectral density (DPSD) distribution of non-line-of-sight (NLOS) paths as a function of UAV altitude. The DPSD surface presents a smooth hill-like morphology, where the overall power level increases monotonically with increasing altitude. This upward trend is most significant near the zero Doppler frequency and relatively gradual at the spectral edges, with the entire surface maintaining continuous and smooth characteristics. This altitude-dependent behavior originates from the synergistic evolution of reflection and scattering paths within the proposed separated modeling framework. It is noteworthy that the spectral shape of NLOS paths remains relatively invariant across different altitudes, exhibiting only overall amplitude elevation.

**Figure 11:**
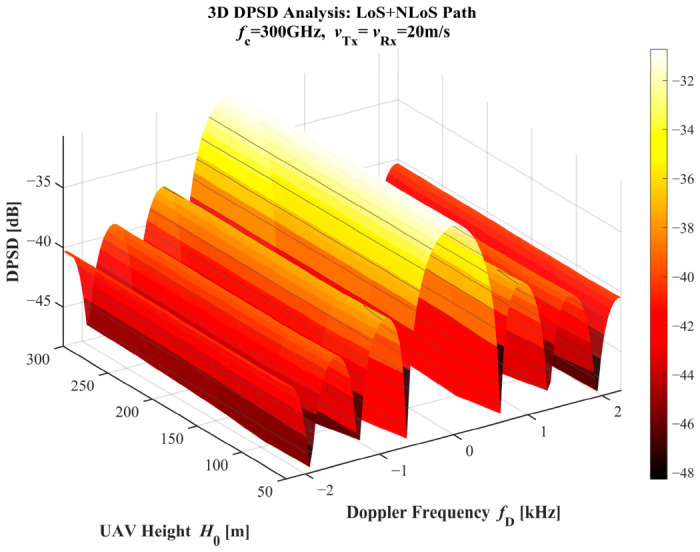
Theoretical results of the DPSD for the LoS + NLoS path with different UAV heights of Tx antenna arrays when the moving time t is 0 s.

Figure 11 illustrates the three-dimensional DPSD characteristics of the combined channel incorporating the line-of-sight (LoS) component, revealing an evolution pattern markedly distinct from the pure NLOS scenario. The most salient feature is the dramatic wave-like undulation structure of the DPSD surface. As altitude increases, the power distribution oscillates periodically between maxima and minima rather than varying monotonically. These oscillations are particularly pronounced near zero Doppler frequency, forming alternating power peaks and valleys that manifest as complex three-dimensional interference patterns across the surface.

This frequency-selective interference phenomenon validates the necessity of separately modeling the LoS, reflection, and scattering paths in the proposed framework. These three propagation mechanisms possess distinct characteristics in both the frequency and spatial domains, and simplified unified modeling approaches are incapable of accurately capturing such intricate interference behavior.

### 5.4. Scattering Cluster Birth-Death Model

**Figure 12:**
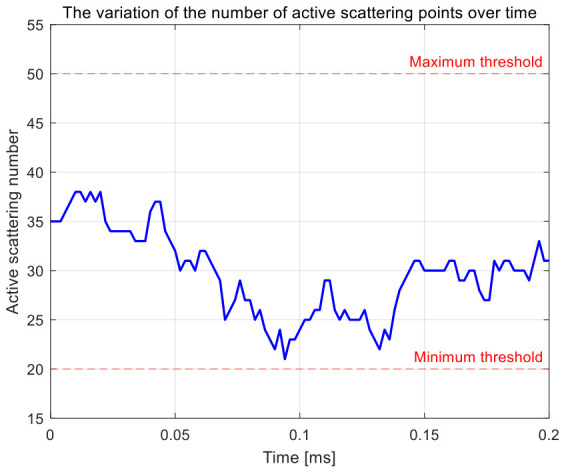
The variation in the number of active scattering points over time.

Figure 12 shows the temporal evolution of the number of active scatterers. The number fluctuates randomly between 20 and 37 over a 0.2 ms time window, with an average of approximately 27. The curve exhibits continuous but irregular variations, with relatively smooth transitions between adjacent time instants. It reflects the natural appearance and disappearance of scatterers in realistic environments. The overall fluctuation range spans over 17 scatterers, consistently remaining within the minimum threshold of 20 and the maximum threshold of 50 (indicated by red dashed lines). It can well demonstrate proper operation of the birth-death control mechanism.

**Figure 13:**
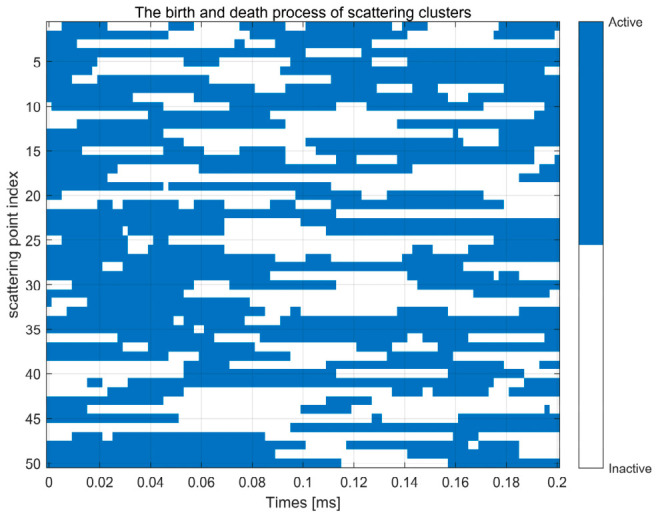
The birth and death process of scattering clusters.

Figure 13 illustrates the spatiotemporal distribution of the scattering cluster birth-death process. In the vertical dimension, scatterers exhibit three typical patterns: some (e.g., indices 5–10) display large continuous blue regions indicating prolonged activity; others show alternating blue-white stripes representing intermittent activity; and others still remain predominantly white, indicating extended inactive periods. In the horizontal dimension, the distribution of active scatterers varies at each time instant, demonstrating clear time-varying characteristics. This dynamic behavior realistically emulates the appearance and disappearance of scatterers in actual wireless propagation environments, such as moving vehicles, pedestrians, and building reflection surfaces.

## 6. Conclusions

In this paper, a comprehensive 3D non-stationary Geometry-Based Stochastic Model (GBSM) is proposed for UAV-to-ground MIMO communication channels. The proposed model establishes a highly dynamic, time-evolving geometric framework to rigorously capture the unique characteristics of UAV-to-ground communications. Specifically, it accounts for the arbitrary 3D high-speed trajectory of the UAV, continuously tracking time-varying velocities and moving directions to accurately reflect severe Doppler non-stationarity. Furthermore, moving beyond the traditional plane-wave approximation, we implement the spherical wavefront assumption to precisely model the non-linear phase variations across the MIMO antenna array elements, which is crucial when interacting with near-field scatterers. Most importantly, to guarantee the spatial consistency of the propagation environment, a dynamic cluster birth-death process is integrated. By incorporating a continuous smoothing weighting function, the model realistically characterizes the gradual blockage and reveal of macroscopic scattering regions (e.g., buildings) as the UAV navigates through complex spaces, successfully eliminating unrealistic discontinuities in channel energy evolution.

Based on this robust theoretical framework, we successfully derived complex closed-form analytical expressions for vital channel statistical properties. Specifically, the Space-Time Correlation Function (STCF) and Doppler Power Spectrum Density (DPSD) were formulated to characterize the time-varying non-stationarity of the channel. Furthermore, extensive Monte Carlo simulations were conducted across various parameter configurations, including scaled antenna arrays and diverse UAV velocities. The perfect agreement between the discrete simulation model and the continuous analytical reference model systematically verifies the mathematical correctness and computational consistency of our derivations.

While this paper establishes a solid mathematical and theoretical foundation for non-stationary UAV channels, future development will focus on the physical validation of the proposed framework. Conducting extensive empirical measurement campaigns and comparative 3D ray-tracing simulations in specific realistic environments (e.g., high-density urban micro scenarios) remains our primary objective for future work.

## Figures and Tables

**Table 1:** Definition of Parameters in the Non-Stationary UAV-MIMO Channel Model.

Symbol	Definition
*D*	Distance on the *y*-axis between Tx and Rx
$h_{Tx},h_{Rx}$	Vertical heights of Tx and Rx
$v_{Tx},v_{Rx}$	Moving velocities of Tx and Rx
φ	Elevation angle between Tx and Rx
$\theta_{LoS,p}^{AoD}(t),\;$ $\phi_{LoS,p}^{AoD}(t)$	Azimuth angle of departure (AAoD) and elevation AoD (EAoD) for the LoS path from the p-th antenna element of Tx to the q-th antenna element of Rx
$\theta_{LoS,q}^{AoA}(t),\;$ $\phi_{LoS,q}^{AoA}(t)$	Azimuth angle of arrival (AAoA) and elevation AoA (EAoA) for the LoS path from the p-th antenna element of Tx to the q-th antenna element of Rx
$\theta_{ref,np}^{AoD}(t),\;$ $\phi_{ref,np}^{AoD}(t)$	AAoD and EAoD for the reflection path from the p-th antenna element of Tx to the n-th reflector, then to the q-th antenna element of Rx
$\theta_{ref,nq}^{AoA}(t),\;$ $\phi_{ref,nq}^{AoA}(t)$	AAoA and EAoA for the reflection path from the p-th antenna element of Tx to the n-th reflector, then to the q-th antenna element of Rx
$\theta_{sca,qm}^{AoD}(t),\;$ $\phi_{sca,qm}^{AoD}(t)$	AAoD and EAoD for the scattering path from the p-th antenna element of Tx to the m-th scatterer, then to the q-th antenna element of Rx
$\theta_{sca,qm}^{AoA}(t),\;$ $\phi_{sca,qm}^{AoA}(t)$	AAoA and EAoA for the scattering path from the p-th antenna element of Tx to the m-th scatterer, then to the q-th antenna element of Rx
$\vartheta_{sca,qm},\vartheta_{ref,nq}$	Random phases of scattering and reflection propagations caused by $S_{m}$ and $R_{n}$, and they can be assumed to be independently, uniformly, and randomly distributed

**Table 2:** Simulation Parameter Data Table.

Symbol	Value
P, Q	2
$\alpha_{Tx},\;$ $\alpha_{Rx},\;$ $\beta_{Tx},\;$ $\beta_{Rx}$	0°
$n_{t,ref},\;$ $n_{t,sca}$	2.2
$\eta_{ref,qp},\;$ $\eta_{ref,q^{\prime }p^{\prime }}$	0.6
$\eta_{sca,qp},\;$ $\eta_{sca,q^{\prime }p^{\prime }}$	0.4
$\sigma_{h,ref}$	0.05 mm
$\sigma_{h,sca}$	0.15 mm

## Data Availability

Data are contained within the article.

## Abbreviations

Abbreviations used in this paper.

**Abbreviation**	**Description**
SAGINs	Space–air–ground–sea integrated networks
MIMO	Multiple-input multiple-output
6G	Sixth generation
UAV	Unmanned aerial vehicle
THz	Terahertz
3D	Three-dimensional
T-ACF	Time autocorrelation function
S-CCF	Space cross-correlation function
DPSD	Doppler power spectrum density
mm Wave	Millimeter wave
A2G	Air-to-ground
A2A	Air-to-air
STC	Space–time correlation
STFC	Space–time–frequency correlation
GBSM	Geometry-based stochastic channel model
Tx	Transmitter
Rx	Receiver
LoS	Line-of-sight
NLoS	Non-line-of-sight
CIR	Channel impulse response
MPCs	Multipath components
